# *Serratia marcescens*: A Versatile Opportunistic Pathogen with Emerging Clinical and Biotechnological Significance

**DOI:** 10.3390/ijms262311479

**Published:** 2025-11-27

**Authors:** Lidia Boldeanu, Mihail Virgil Boldeanu, Marius Bogdan Novac, Mohamed-Zakaria Assani, Lucrețiu Radu

**Affiliations:** 1 Department of Microbiology, Faculty of Medicine, University of Medicine and Pharmacy of Craiova, 200349 Craiova, Romania; lidia.boldeanu@umfcv.ro; 2 Department of Immunology, Faculty of Medicine, University of Medicine and Pharmacy of Craiova, 200349 Craiova, Romania; mihail.boldeanu@umfcv.ro; 3 Department of Anesthesiology and Intensive Care, Faculty of Medicine, University of Medicine and Pharmacy of Craiova, 200349 Craiova, Romania; 4 Doctoral School, University of Medicine and Pharmacy of Craiova, 200349 Craiova, Romania; 5 Department of Hygiene, Faculty of Medicine, University of Medicine and Pharmacy of Craiova, 200349 Craiova, Romania; lucretiu.radu@umfcv.ro

**Keywords:** *Serratia marcescens*, prodigiosin, virulence, nosocomial infections, biofilm, antibiotic resistance, biotechnology

## Abstract

*Serratia marcescens* is a Gram-negative opportunistic pathogen renowned for its extensive ecological versatility and clinical significance. Once considered a benign saprophyte, it has now been recognized as a notable etiological agent in nosocomial infections, predominantly affecting immunocompromised hosts. Its pathogenicity is mediated by an array of virulence determinants, including hemolysins, proteolytic enzymes, siderophores, and the biosynthesis of the pigmented secondary metabolite prodigiosin, which exhibits notable anticancer and immunomodulatory activities. *S. marcescens* exhibits proficient biofilm-forming capabilities that underpin persistent device colonization and confer resilience against antimicrobial therapies. Beyond its clinical impact, *S. marcescens* is of interest in industrial biotechnology and environmental bioremediation applications. This comprehensive review delineates current insights into its taxonomy, virulence pathways, antimicrobial resistance mechanisms, and emerging biotechnological utilities, emphasizing the dual challenges and opportunities it presents to microbiology and therapeutic development.

## 1. Introduction

*Serratia marcescens* (*S. marcescens*) is a Gram-negative, facultative anaerobic bacillus belonging to the Yersiniaceae family within the Enterobacterales order. First described in 1819 by Bartolomeo Bizio, it attracted attention due to its production of a striking red pigment, later identified as prodigiosin. Once regarded as a benign saprophyte or laboratory contaminant, *S. marcescens* is now recognized as a significant opportunistic pathogen, particularly in the context of healthcare-associated infections (HAIs) [1,2,3,4].

Its remarkable environmental adaptability allows it to thrive in various ecological niches, including water, soil, plants, insects, and hospital surfaces. This widespread presence is partly due to its ability to survive in low-nutrient environments and resist common disinfectants, which facilitates its colonization of medical devices such as catheters and ventilators [5]. In clinical settings, *S. marcescens* is linked to urinary tract infections, bloodstream infections, respiratory infections, wound infections, and meningitis, with a particular tendency to affect immunocompromised patients, neonates, and populations in intensive care units (ICUs) [6,7]. Beyond its clinical significance, *S. marcescens* has attracted attention in biotechnology and environmental microbiology. The red pigment prodigiosin, for example, has shown antimicrobial, antimalarial, anticancer, and immunomodulatory properties [8,9].

Despite its biotechnological potential, the emergence of multidrug-resistant (MDR) and extensively drug-resistant (XDR) *S. marcescens* strains has become a significant global concern [10]. This review aims to offer a comprehensive overview of *S. marcescens*, focusing on its molecular pathogenicity, clinical significance, antibiotic resistance, and possible biotechnological applications.

## 2. Taxonomy and General Characteristics

*S. marcescens* was first investigated in 1819 by the Venetian pharmacist Bartolomeo Bizio during outbreaks of red discoloration on polenta in Padua. Bizio introduced the binomen a few years later, choosing the genus name to honor Serafino Serrati and the epithet marcescens from Latin, “to decay,” reflecting the unstable red pigment then observed [11,12].

Nomenclature shifted repeatedly through the late nineteenth and early twentieth centuries, with the organism treated by various authors as Bacillus prodigiosus, or Chromobacterium prodigiosum. Bizio’s original combination was progressively restored as taxonomic concepts stabilized, and *S. marcescens* is the type species of Serratia. In 1961, Martinec and Kocur proposed ATCC 13,880 as the neotype, consolidating usage across culture collections. The name appears on the Approved Lists of Bacterial Names, 1980 [13,14].

Genome-based phylogeny now assigns Serratia to the order *Enterobacterales*, family *Yersiniaceae*, following the 2016 reorganization by Adeolu and colleagues. This split the former Enterobacteriaceae sensu lato into several families on the basis of conserved signature indels and multilocus phylogeny, a scheme adopted by LPSN and subsequent reviews [15,16].

Clinically relevant strains are often non-pigmented, while environmental isolates frequently produce prodigiosin, the historical source of confusion with “bloody” foods and surfaces in early reports [17].

*S. marcescens* is a facultatively anaerobic, motile, Gram-negative bacillus, usually measuring 0.5–0.8 µm in width and 0.9–2.0 µm in length. It belongs to the phylum *Pseudomonadota*, class *Gammaproteobacteria*, order *Enterobacterales*, and is currently classified in the family *Yersiniaceae*, following recent taxonomic updates based on genomic phylogeny [18].

Initially classified as part of the family Enterobacteriaceae, *S. marcescens* was reclassified into *Yersiniaceae* due to significant differences in 16S rRNA gene sequences and genome-wide comparative studies. The genus *Serratia* comprises over 20 species, but *S. marcescens* remains the most clinically significant and extensively studied member [19].

*S. marcescens* exhibits robust growth on standard laboratory media, including nutrient agar and tryptic soy agar. The colonies typically present as moist and smooth, with coloration ranging from red to off-white, influenced by pigment synthesis and incubation conditions. Prodigiosin production is generally inhibited at 37 °C and reaches peak levels between 25–30 °C [20]. On blood agar, colonies are usually gray and non-hemolytic, though hemolysis may occur in some strains [21,22]. This thermally regulated pigmentation mechanism may elucidate why clinical isolates adapted to human core temperatures frequently exhibit lack of pigmentation. Additionally, its environmental resilience allows it to persist in varied and nutrient-scarce conditions, such as exposure to disinfectants, saline solutions, and chlorinated water [23]. An important advancement in media options is CHROMagar™-Serratia (CHROMagar Paris, France), a selective chromogenic medium engineered for the detection and isolation of *S. marcescens*. It efficiently suppresses a wide range of competing microorganisms [24].

## 3. Virulence Factors

**Figure 1:**
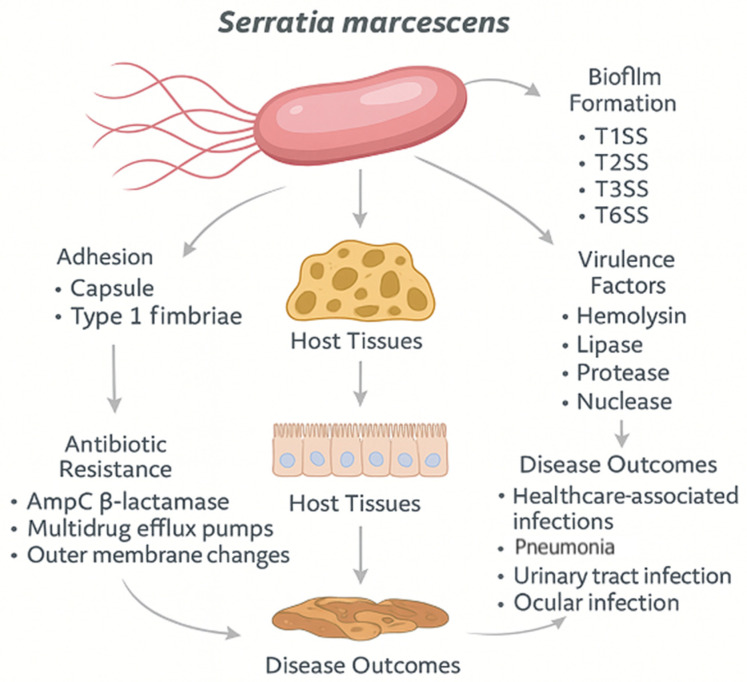
Overview of *S. marcescens* pathogenic mechanisms and clinical impact (Figure created in Canva, https://www.canva.com). The image emphasizes the multifactorial pathogenesis of *S. marcescens*, highlighting the interaction between virulence, resistance, and clinical persistence: Adhesion mechanisms (left side): Initial colonization is mediated by type I fimbriae and, in some strains, a thin capsule, which enables attachment to host surfaces and abiotic materials (e.g., catheters). This step is crucial for subsequent biofilm development and immune evasion. Biofilm formation (right side): The bacterium forms biofilms through coordinated activity of Type I, II, III, and VI Secretion Systems (T1SS–T6SS). These biofilms protect the community from antibiotics and host immunity, especially on medical devices. Virulence factors (right side): *S. marcescens* produces a range of extracellular enzymes, including hemolysin, lipase, protease, and nuclease, that aid in tissue invasion, immune modulation, and nutrient acquisition. Antibiotic resistance mechanisms (bottom left): Clinical isolates often display intrinsic and acquired resistance, including AmpC β-lactamases, multidrug efflux pumps, and modifications of outer membrane permeability (e.g., porin loss). Host tissue interaction and clinical outcomes (bottom): After adhesion and invasion, the pathogen interacts with epithelial and immune barriers, leading to healthcare-associated infections, such as ventilator-associated pneumonia, urinary tract infections, ocular infections, and catheter- and device-associated bloodstream infections.

*S. marcescens* possesses a diverse arsenal of virulence factors that enable colonization, tissue invasion, immune evasion, and persistence in both environmental and host-associated niches. These virulence determinants include extracellular enzymes, pigments, adhesion molecules, secretion systems, and iron acquisition mechanisms (Figure 1). The coordinated regulation of these factors contributes significantly to the bacterium’s pathogenic potential, particularly in healthcare-associated infections.

*S. marcescens* deploys a coordinated set of factors that act on host cells, competitors, and surfaces. The pore-forming hemolysin ShlA is secreted and activated via its cognate partner ShlB through a type Vb two-partner pathway, and ShlB-dependent conformational activation of ShlA has been shown genetically and biochemically [25,26,27]. A second hemolytic activity is mediated by the secreted phospholipase PhlA, which generates lysophospholipids and causes contact-dependent hemolysis on blood agar [21]. Extracellular metalloproteases of the serralysin family contribute to tissue injury and inflammation, including induction of IL-6 and IL-8 in airway epithelial cells, and belong to the RTX protease group with stabilizing interactions between their domains [28,29]. Iron acquisition relies on catecholate siderophores such as serratiochelin, which are required for full virulence during bloodstream infection in a clinical isolate background [30]. Adhesion and biofilm formation depend on type 1 fimbriae under control of the cAMP–CRP regulatory system. *S. marcescens* is implicated in the biofilm formation [31,32]. Motility on surfaces is aided by lipopeptide biosurfactants called serrawettins, including serratamolide, which promote swarming and can be hemolytic [33,34]. Antibacterial antagonism is mediated by a conserved type VI secretion system that delivers pore-forming effectors, with Ssp6 forming cation-selective pores that depolarize target cells and the recently described Ssp4 representing a second pore-forming effector in the same system [35,36]. These modules are coordinated by N-acyl homoserine lactone quorum sensing of the LuxIR type, which regulates adhesion, exoenzymes, motility, hemolysis, and biofilm traits, with surface-dependent control documented in strain MG1 [37].

*Hemolysins and Cytotoxins*-One of the key virulence determinants are ShlA, a pore-forming hemolysin secreted via the ShlB secretion system. ShlA lyses erythrocytes, epithelial cells, and immune cells, promoting nutrient acquisition and tissue damage [26]. ShlA expression is tightly regulated by quorum sensing and the Rcs phosphorelay system. Additionally, *S. marcescens* produces PhlA, a phospholipase-like hemolysin with cytotoxic effects on host cells and bactericidal activity against competing microbes [21].

*Extracellular Enzymes*-*S. marcescens* secretes a variety of hydrolytic enzymes, including [21,29,38,39,40,41,42]:

- Proteases (e.g., serralysin): Degrade host tissue components and immune mediators such as immunoglobulins and complement proteins.
- Lipases and phospholipases: Involved in membrane disruption and nutrient liberation.
- DNases and nucleases: Facilitate evasion of neutrophil extracellular traps (NETs).

These enzymes are often encoded on pathogenicity islands and can be upregulated in biofilm-forming strains [43].

*Prodigiosin*-Prodigiosin is a red, tripyrrole pigment produced by many *S. marcescens* strains. While not essential for pathogenicity, it contributes to oxidative stress resistance, promotes bacterial survival, exhibits cytotoxicity against cancer cells, and has immunomodulatory activity [9]. In polymicrobial environments, prodigiosin may function as a competitive advantage molecule, exhibiting antimicrobial activity against other microbes [8].

*Secretion Systems*-*S. marcescens* harbors multiple secretion systems that mediate virulence [25,44,45,46,47]:

- Type I Secretion System (T1SS): Transports hemolysin (ShlA).
- Type III Secretion System (T3SS): Injects effector proteins into host cells (limited distribution in clinical strains).
- Type IV Secretion System (T4SS): Implicated in horizontal gene transfer and virulence gene dissemination.
- Type VI Secretion System (T6SS): Functions in bacterial competition and delivery of toxins to eukaryotic cells. The T6SS is particularly active in biofilm and polymicrobial environments. The Type VI Secretion System is especially crucial in interbacterial antagonism, particularly in polymicrobial environments. T6SS in *S. marcescens* is encoded by hcp/vgrG loci and is closely regulated by quorum sensing and stress responses [48].

*Adhesins and Fimbriae*-Adherence to host tissues and abiotic surfaces is mediated by: type 1 fimbriae, curli-like fimbriae, and outer membrane proteins (OMPs). These structures are crucial for the colonization of urinary catheters and respiratory devices, representing a critical step in the initiation of biofilms. Disruption of fimA significantly decreases adherence and biofilm biomass [31]. Along with the cAMP–CRP pathway, surface attachment in *S. marcescens* is also controlled by OxyR, a redox-sensitive transcriptional regulator that influences genes involved in fimbrial expression and early biofilm formation [49]. Cyclic AMP levels in *S. marcescens* are dynamically regulated by cAMP-phosphodiesterase activity, which in turn influences type I fimbriae expression and biofilm development, emphasizing the role of second messenger signaling in surface colonization [32].

*Iron Acquisition Systems*-Iron is essential for bacterial metabolism and survival, especially during infection. *S. marcescens* produces multiple siderophores, including enterobactin and serratiochelin (unique to Serratia). These high-affinity iron chelators facilitate survival in iron-limited environments, such as within the host, and are essential for full virulence [50].

*Quorum Sensing and Regulation*-Virulence in *S. marcescens* is modulated by quorum sensing via N-acyl homoserine lactones (AHLs). The SmaI/SmaR system controls the expression of biofilm genes, proteases, pigment biosynthesis, and motility. Disruption of quorum sensing results in reduced virulence and biofilm formation, making it a potential therapeutic target [51].

These mechanisms show the versatility of *S. marcescens* in both acute and persistent infections, and they serve as potential targets for anti-virulence therapeutic strategies.

## 4. Biofilm Formation

Biofilm formation is a key virulence strategy used by *S. marcescens*, allowing it to survive hostile environments, evade host immunity, and withstand antimicrobial treatments. Biofilms are organized communities of bacterial cells surrounded by a self-produced extracellular polymeric substance (EPS) that attaches to both living and non-living surfaces [52]. In healthcare environments, *S. marcescens* biofilms are particularly associated with medical devices, including urinary catheters, central venous lines, prosthetic valves, and ventilator tubes [53].

*S. marcescens* develops surface-associated communities through a regulated process of adhesion, maturation, and dispersal driven by nutrient sensing, quorum signaling, and global regulators. Type 1 fimbriae are central to initial adhesion and are controlled by the cyclic AMP-dependent catabolite repression system, which modulates fimbrial expression and biofilm development in response to carbon source availability [31,54].

Quorum sensing via N-acyl homoserine lactones coordinates adhesion, maturation, and sloughing dynamics in model strains such as MG1 [37]. Lipopeptide biosurfactants from the serrawettin family, such as serratamolide, promote surface motility that is coupled to the initial stages of biofilm development and may contribute to hemolytic activity [34]. The matrix contains extracellular DNA, and DNase activity alters adhesion and dispersal, supporting a functional role for eDNA in Serratia biofilms [55]. Envelope-stress signaling through the Rcs phosphorelay system intricately regulates biofilm-associated gene expression, as demonstrated by transcriptomic analyses of clinical isolates harboring mutations in GumB, an inner-membrane Rcs regulatory component [56].

Recent research delineates the mechanistic linkage between motility and biofilm maturation in Serratia marcescens. A 2025 publication demonstrates that the metalloprotease PrtA is critical for effective biofilm formation by facilitating flagellar turnover; its proteolytic function is indispensable. Loss of prtA results in thinner, less viable biofilms, which can be rescued through complementation with wild-type PrtA or exogenous enzyme supplementation. These findings position extracellular proteolysis as a key factor bridging motility suppression and the development of mature extracellular matrix architecture [57]. In parallel, a recent study delineates the genomic and phenotypic factors underlying Serratia’s interactions with plants, emphasizing colonization determinants, secretion system components, and quorum sensing mechanisms that facilitate surface attachment behaviors pertinent to biofilm development within environmental niches [58].

*Stages of Biofilm Development*—biofilm formation in *S. marcescens* follows the classic five-stage model [59]:

- Initial attachment—mediated by flagella, fimbriae, and surface adhesins.
- Irreversible attachment—involving enhanced EPS production and expression of specific biofilm genes.
- Maturation I and II—3D architecture develops, with water channels and nutrient gradients.
- Dispersion—cells detach and colonize new surfaces.

Flagella play a dual role by promoting motility during early attachment and contributing to dispersal in mature biofilms [51].

*EPS*—the EPS matrix comprises polysaccharides, proteins, lipids, and extracellular DNA (eDNA). It serves multiple purposes: providing mechanical stability, retaining nutrients, protecting against antibiotics and host immune responses, and facilitating horizontal gene transfer. *S. marcescens* produces cellulose and biofilm-associated proteins (Bap-like proteins) that contribute to EPS integrity [31].

*Quorum Sensing in Biofilm Regulation*—biofilm development is tightly controlled by quorum sensing (QS), a cell-density-dependent signaling system mediated by AHLs. The SmaI/SmaR system regulates: EPS synthesis, motility, enzyme secretion, and prodigiosin production. Mutations in smaI or smaR significantly reduce biofilm biomass, confirming their regulatory role [51].

*Resistance Mechanisms in Biofilms*—Biofilm-related resistance arises through restricted antibiotic penetration, altered metabolic states (dormant persister cells), upregulation of efflux pumps, and enhanced expression of stress-response genes. These mechanisms make *S. marcescens* biofilms particularly resilient and difficult to target using conventional antimicrobial strategies [60].

**Figure 2:**
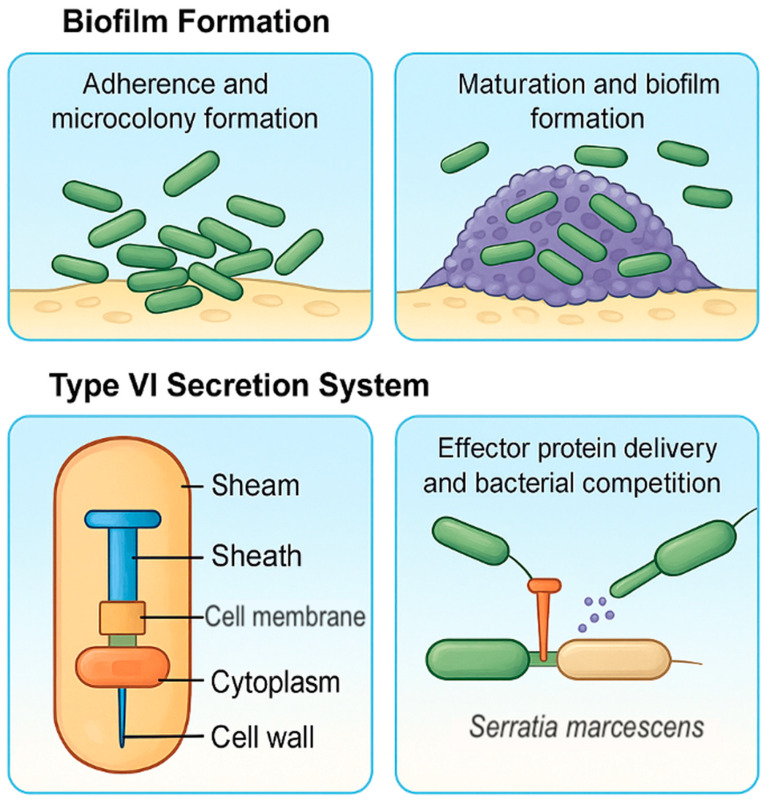
Biofilm Development and Type VI Secretion System (T6SS) Function in *S. marcescens* (Figure created in Canva, https://www.canva.com). This figure highlights two key virulence processes in *S. marcescens*: biofilm development and T6SS, which are essential for surface colonization and interbacterial competition.

Biofilm and T6SS are functionally connected (Figure 2); the T6SS may kill competitors during early biofilm stages, thereby helping to establish clonal dominance [61]. T6SS is essential for surface colonization by enabling competitive exclusion within polymicrobial communities commonly found on medical devices. On non-living surfaces like catheters and endotracheal tubes, *S. marcescens* uses T6SS to kill neighboring Gram-negative bacteria through contact-dependent delivery of toxic effector proteins (e.g., phospholipases, nucleases). This promotes clonal growth, biofilm stability, and niche dominance, particularly during the early stages of colonization. T6SS activity is often increased under stress conditions common in medical settings (e.g., limited nutrients, host antimicrobials) and may be linked to quorum sensing and biofilm development pathways [45,62,63].

Top Panel—Biofilm Formation. Left (Adherence and Microcolony Formation): Free-living planktonic cells initiate surface attachment via fimbriae and adhesins. Early adhesion leads to the formation of small microcolonies—a crucial first step in biofilm development. Right (Maturation and Biofilm Formation): As bacterial communities grow, they become encased in an extracellular polymeric substance (EPS) made of polysaccharides, proteins, and eDNA. This mature biofilm protects against antibiotics and immune responses.

Bottom Panel—T6SS. Left (Structural Diagram): T6SS resembles an inverted bacteriophage tail anchored in the bacterial membrane. The complex includes a sheath (a contractile structure that drives the injection apparatus outward), an internal tube for effector delivery, and the cell membrane, cytoplasm, and wall (the structural context of the injection system within the bacterium). Right (Effector Delivery): *S. marcescens* deploys T6SS to inject toxic effector proteins into neighboring bacteria. This promotes bacterial competition, niche dominance, and survival in polymicrobial environments such as biofilms or host tissues.

## 5. Antibiotic Resistance

Resistance reflects intrinsic, acquired, and adaptive components. Chromosomal AmpC β-lactamase is inducible, with derepression arising via mutations in the AmpR–AmpD–AmpG network under β-lactam exposure, which elevates cephalosporin MICs and undermines third-generation cephalosporins [64,65]. Intrinsic resistance to polymyxins is characteristic of *S. marcescens* and is mediated by lipid A modification pathways, notably arnBCADTEF and related enzymes that add 4-amino-4-deoxy-L-arabinose or phosphoethanolamine to lipid A, decreasing colistin binding [4,66]. Efflux systems contribute broadly, including RND pumps SdeAB, SdeXY, and SdeCDE that export multiple classes, and the ABC pump MacAB, which protects against oxidative stress and enhances tolerance to polymyxins and aminoglycosides [67,68,69]. Outer-membrane permeability shifts, for example, reduced OmpF or OmpC porins, add to carbapenem and cephalosporin resistance when combined with β-lactamases [70].

**Figure 3:**
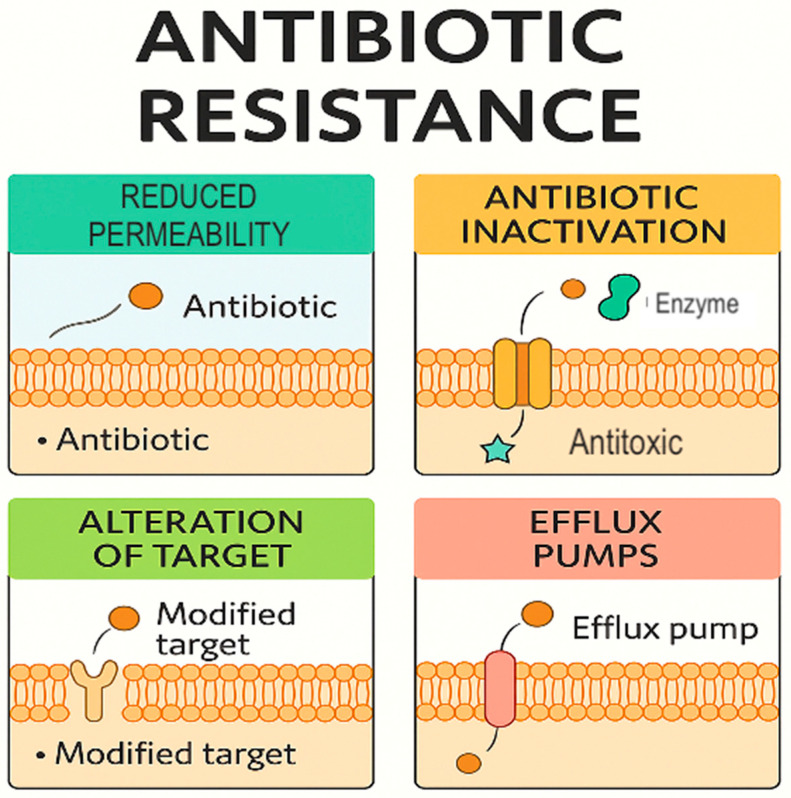
Mechanisms of antibiotic resistance in *S. marcescens* (Figure created in Canva, https://www.canva.com). This diagram illustrates the four main mechanisms by which bacteria—especially Gram-negative species like *S. marcescens*—avoid the effects of antibiotics: 1. Reduced permeability—Some antibiotics rely on porin channels to enter bacterial cells. In *S. marcescens*, downregulation or mutation of outer membrane proteins, such as OmpF, decreases membrane permeability, reducing intracellular antibiotic levels. This mechanism mainly affects β-lactams and carbapenems. 2. Antibiotic inactivation—bacteria produce enzymes that break down or modify antibiotics chemically. *S. marcescens* produces AmpC β-lactamase (chromosomal) and can acquire ESBLs or carbapenemases (e.g., KPC, NDM), which hydrolyze β-lactam antibiotics, rendering them ineffective. 3. Target alteration—Resistance can develop from mutations in target enzymes like DNA gyrase (gyrA) or topoisomerase IV (parC), decreasing the binding of fluoroquinolones. Target site protection or modifications of ribosomes may also occur, depending on the antibiotic class. 4. Efflux pumps—Multidrug resistance is often caused by efflux systems such as the SdeAB-RND and SdeXY-TolC pumps in *S. marcescens*. These transporters expel antibiotics from the cell before they can reach inhibitory levels, leading to resistance against tetracyclines, fluoroquinolones, and chloramphenicol.

Its intrinsic and acquired resistance mechanisms pose a serious challenge in clinical management (Figure 3), especially for immunocompromised patients and those in ICUs.

*Intrinsic Resistance*—*S. marcescens* exhibits natural resistance to several antibiotics, including ampicillin, first-generation cephalosporins, macrolides, and colistin (in many isolates). This intrinsic resistance is primarily due to low outer membrane permeability, efflux pump systems (e.g., SdeAB, SdeXY-TolC), and the chromosomally encoded inducible AmpC β-lactamase, which hydrolyzes penicillins and early cephalosporins [4,71,72].

*Acquired Resistance Mechanisms*—clinical isolates increasingly harbor mobile genetic elements, including plasmids, transposons, and integrons, carrying resistance genes for [73,74,75]:

- Extended-spectrum β-lactamases (ESBLs)
- Carbapenemases, e.g., KPC, NDM, VIM;
- Aminoglycoside-modifying enzymes;
- Fluoroquinolone resistance.

*Resistance in Biofilms*—biofilm-associated *S. marcescens* shows dramatically increased resistance to antibiotics, even when planktonic cells are susceptible. Mechanisms include: reduced penetration of antibiotics, altered metabolic activity (e.g., persister cells), and upregulation of efflux pumps and stress-response genes. Biofilm cells may require 100–1000× higher antibiotic concentrations for eradication compared to planktonic cells [60].

*Clinical Impact*—infections caused by MDR *S. marcescens* are associated with: higher morbidity and mortality, increased hospitalization duration, and limited therapeutic options. Particularly concerning are carbapenem-resistant strains, which the World Health Organization (WHO) and Centre for Disease Control and Prevention (CDC) classify as critical priority pathogens [76].

*Therapeutic strategies* for MDR *S. marcescens* include carbapenems (if susceptibility is confirmed), fourth-generation cephalosporins (e.g., cefepime), fluoroquinolones, combination therapy (e.g., β-lactam + aminoglycoside), and new agents such as ceftazidime-avibactam, cefiderocol, or tigecycline, depending on the resistance profile [7]. However, colistin—used as a last resort—often shows poor activity against *S. marcescens*, either due to intrinsic resistance or adaptive modifications of lipid A [77].

## 6. Environmental and Industrial Aspects. Industrially Relevant Enzymes from *S. marcescens*

Biotechnologically, *S. marcescens* is utilized to produce enzymes such as chitinase, protease, lipase, and DNase for applications in agriculture, food processing, and bioremediation [78,79]. Some strains promote plant growth and suppress pathogens through antifungal and nematicidal actions [80]. It can also degrade hydrocarbons, textile dyes, and absorb heavy metals, making it valuable in wastewater treatment [81].

**Figure 4:**
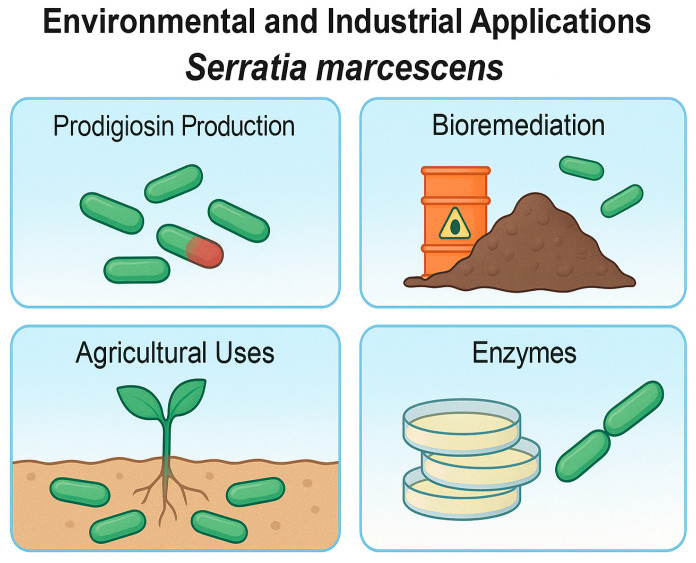
Environmental and industrial applications of *S. marcescens* (Figure created in Canva, https://www.canva.com). This infographic summarizes the leading biotechnological roles of *S. marcescens* in non-clinical settings: 1. Prodigiosin production: *S. marcescens* synthesizes prodigiosin, a red tripyrrole pigment with antimicrobial, anticancer, and immunosuppressive properties. It has been investigated for pharmaceutical applications, natural dye production, and bioelectronic devices due to its ability to shuttle electrons. 2. Bioremediation: environmental strains of *S. marcescens* can degrade organic pollutants (e.g., hydrocarbons, textile dyes) and absorb heavy metals, making them useful in wastewater treatment, soil restoration, and detoxification of toxic compounds. 3. Agricultural uses: certain strains act as plant growth-promoting rhizobacteria (PGPR). They produce chitinases, siderophores, and antifungal metabolites that protect crops from pathogens and enhance nutrient uptake—especially in sustainable or organic agriculture. 4. Enzyme production: *S. marcescens* is a rich source of industrial enzymes such as proteases (e.g., serralysin-like), chitinases (for fungal control), lipases, and DNases, which are used in biomedicine, the food industry, textile processing, and biocontrol products.

Figure 4 summarizes the leading biotechnological roles of *S. marcescens* in non-clinical settings.

*S. marcescens* supplies an enzyme toolbox already used in biotechnology, with chitinases, metalloproteases, and lipases as the main workhorses. For chitin valorization, *S. marcescens* encodes multiple family 18 chitinases, classically ChiA, ChiB, and ChiC, which act synergistically on crystalline substrates and can be cloned and expressed in safe hosts. These enzymes convert crustacean shell waste into N-acetylglucosamine and chitooligosaccharides for food, agriculture, and materials pipelines, and production can be raised by medium optimization or by engineering carbohydrate-binding modules for better attack on crystalline chitin [82,83,84,85,86].

Serratia metalloproteases of the serralysin family include serratiopeptidase, which has long been formulated as a therapeutic enzyme and evaluated as an anti inflammatory, mucolytic, and antibiofilm adjunct. Recent bioprocess work focuses on higher titers in nonpathogenic hosts, secretion tags, and downstream polishing compatible with pharmaceutical specifications. The same catalytic traits are attractive in industrial cleaning and food processing, provided the enzyme is produced in a qualified chassis [87,88,89].

The enzymatic profile of *S. marcescens* shows significant industrial value, with applications in agriculture, biotechnology, and medicine. This species produces various extracellular enzymes, including chitinase, serralysin-like protease, lipase, and DNase, each with unique substrate specificities and optimal conditions [90]. Several enzymes possess beneficial traits, such as tolerance to solvents or alkaline pH, making them suitable for demanding industrial processes [91]. Notably, *S. marcescens* strain E-15 produces serratiopeptidase, a proteolytic enzyme known for its anti-inflammatory effects in medical products [87]. Additionally, the organism synthesizes the red pigment prodigiosin through a dedicated biosynthetic pathway, with promising uses in oncology, antimicrobial coatings, and bioelectronics [79]. This diverse enzymatic arsenal highlights *S. marcescens*’s role as both a valuable biotechnological resource and a potential clinical concern, stressing the importance of careful strain selection and biosafety evaluations in practical applications.

Recent work broadens the clinical and industrial scope of prodigiosin. Two 2024 reviews synthesize bioactivity portfolios and manufacturing routes, from antimicrobial and antitumor effects to process and genetic strategies that raise yield and purity, with attention to stability and formulation constraints. A 2024 primary study characterizes prodigiosin from an *S. marcescens* clinical isolate by spectroscopy and chromatography, and reports in vitro antibacterial, antifungal, and anticancer activities supported by docking analyses. Together, these studies support the dual perspective developed here, clinical risk from a pigment-producing opportunist, and translational potential where standardized production and safety testing are feasible [79,92,93].

WHO guidance relevant to *S. marcescens* frames it within Enterobacterales priorities. The 2024 WHO Bacterial Priority Pathogens List designates carbapenem-resistant Enterobacterales as critical priority, and third-generation cephalosporin-resistant Enterobacterales as high priority, to steer research and development, and policy focus [94,95]. WHO’s AWaRe program and the AWaRe Antibiotic Book emphasize optimizing use of Access agents, restricting Reserve agents to confirmed or strongly suspected multidrug-resistant infections, and set a country target that at least 60 percent of antibiotic consumption be Access group medicines [96].

## 7. Clinical Applications and Innovative Therapies

Recent advances in microbiology and infectious disease management have led to the development of new treatment options for combating drug-resistant *S. marcescens* infections, which extend beyond traditional antibiotics. This section highlights promising clinical innovations:

- *Bacteriophage Therapy*: Specific lytic phages targeting *S. marcescens* have shown effectiveness in disrupting biofilms and working together with antibiotics. Experimental and compassionate-use cases demonstrate clinical benefits in treating difficult-to-treat device-related infections and chronic wounds [97].
- Phage-Antibiotic Synergy (PAS): Using phages with sub-inhibitory amounts of antibiotics (such as ciprofloxacin, cefepime) can improve bacterial elimination and slow resistance development. This synergy is especially useful in treating biofilm-related infections where monotherapy often fails [98].
- *CRISPR* (*Clustered Regularly Interspaced Short Palindromic Repeats*) *and Cas* (*CRISPR-associated proteins*): Synthetic CRISPR constructs delivered through conjugative plasmids or engineered phages are being tested to selectively eliminate resistance genes in Enterobacterales. Although not yet approved for clinical use, preclinical studies have shown success in making MDR strains of *S. marcescens* more sensitive to standard antibiotics [99].
- *Anti-Virulence Strategies*: Small-molecule inhibitors of the T6SS, QS, and biofilm matrix synthesis are being developed. Instead of directly killing bacteria, these agents aim to disarm pathogens, reducing host damage and the selective pressure for resistance [62,100].
- *Nanoparticle-Based Delivery*: Nanocarriers, such as liposomes, dendrimers, and silver nanoparticles, can be utilized to deliver antibiotics or phages directly to biofilm-embedded *S. marcescens*, thereby increasing local drug concentration and overcoming matrix barriers [101].

Plant essential oils and phenolic phytochemicals show anti-Serratia and anti-biofilm activity in vitro, acting as adjuvants rather than replacements for antibiotics. Thymol, carvacrol, and eugenol, and extracts from spices such as cinnamon, clove, and garlic, inhibit growth, reduce quorum sensing outputs such as prodigiosin production, and disrupt early biofilm formation. These effects align with anti-virulence strategies by attenuating adhesion, motility, pigment regulation, and matrix stability, and they may enhance antibiotic activity when used in combinations. Evidence comes from controlled in vitro studies showing that selected essential oils and their principal components suppress *S. marcescens* growth and quorum-regulated pigment production, and that eugenol reduces multiple quorum-regulated functions. Translation is limited by variability in composition, dosing, and delivery, and by the lack of standardized clinical data, therefore these agents are best considered as investigational adjuvants that could be combined with antibiotics or device-care measures [51,102].

These approaches represent a shift toward precision and adjuvant therapy, tailored to overcome the adaptive defenses of this versatile pathogen.

## 8. Current Challenges and Future Directions

*S. marcescens* is a healthcare-associated opportunist that colonizes moist environments and devices, then causes infection when host defenses are impaired or barriers are breached. Reported syndromes include urinary tract infection, pneumonia including ventilator-associated pneumonia, bloodstream infection often catheter related, surgical site and intra-abdominal infections, biliary infection, and less commonly endocarditis and skin or soft-tissue infection [103,104].

In neonates and very preterm infants, *S. marcescens* is a well-described cause of late-onset sepsis and meningitis, with repeated NICU outbreaks linked to environmental reservoirs such as sinks and wet surfaces. Incidence estimates around 2.3 late-onset infections per 1000 very preterm infants have been reported, and mortality during outbreaks can be high [6,105,106].

Ocular disease ranges from conjunctivitis to sight-threatening keratitis, most often in contact lens wearers, supported by experimental work showing strong adherence of *S. marcescens* to lens materials [107,108].

Several challenges persist in the clinical management and biotechnological application of *S. marcescens*. The rapid proliferation of MDR and XDR strains, especially those resistant to carbapenems, highlights the need for improved surveillance and the development of new therapies. New antibiotics, such as cefiderocol, show potential but may encounter emerging resistance [109,110].

Biofilm-related tolerance complicates treatment; future strategies include anti-biofilm coatings and quorum-sensing inhibitors [60]. The T6SS has become a target for anti-virulence approaches [111]. Bacteriophage therapy and CRISPR-based techniques are being studied for precise targeting of resistance and virulence genes [99,112,113].

Whole-genome sequencing helps track environmental sources and prevent outbreaks [114,115]. Meanwhile, biotechnology should be restricted to non-pathogenic strains to ensure biosafety.

Omics-Based Insights in the Biology of *S. marcescens*.

High throughput proteomics and proteogenomics have refined how we view adaptation, virulence, and resistance in *S. marcescens*. Under meropenem exposure, multidrug-resistant isolates remodel the abundance of proteins linked to cell envelope maintenance, stress responses, efflux, and central metabolism, a pattern consistent with antibiotic survival programs and tolerance phenotypes. Baseline proteomic mapping with high resolution mass spectrometry has cataloged secreted enzymes, transporters, and factors implicated in pathogenicity and multi drug resistance, offering a reference dataset for comparative analyses across growth conditions and strain backgrounds. A proteogenomic subtractive workflow further prioritized non homologous essential proteins, predicted druggability and localization, and highlighted candidates for target discovery and vaccine design in *S. marcescens*. These proteomic and proteogenomic datasets complement transcript level studies and metabolite profiling by supplying mechanistic readouts at the protein level, identifying pathways that shift under antibiotic pressure, and proposing experimentally testable targets or biomarkers. Together, they support a systems perspective that links resistance mechanisms in Section 5 and biofilm behavior in Section 4 to specific regulons, enzymes, and transport systems that can be measured and perturbed [116,117,118].

## 9. Conclusions

*S. marcescens* demonstrates the dual nature of bacteria, serving as both a clinically important opportunistic pathogen and a valuable biotechnological tool. Its ability to resist antibiotics, form biofilms, and withstand environmental conditions requires careful monitoring and infection control.

At the same time, its production of bioactive metabolites and enzymes offers value for medical, agricultural, and environmental uses. Future strategies should focus on developing safe strains for industrial applications, targeted antimicrobial treatments, and ongoing genomic surveillance to balance biosafety with biotechnological progress.

## Data Availability

No new data were created or analyzed in this study. Data sharing is not applicable to this article.

## Abbreviations

The following abbreviations are used in this manuscript:

HAIs	healthcare-associated infections
ICUs	intensive care units
MDR	multidrug-resistant
XDR	extensively drug-resistant
NETs	neutrophil extracellular traps
T1SS	Type I Secretion System
T3SS	Type III Secretion System
T4SS	Type IV Secretion System
T6SS	Type VI Secretion System
OMPs	outer membrane proteins
AHLs	N-acyl homoserine lactones
EPS	extracellular polymeric substance
QS	quorum sensing
eDNA	extracellular DNA
ESBLs	Extended-spectrum β-lactamases
CRISPR	Clustered Regularly Interspaced Short Palindromic Repeats
CDC	Disease Control and Prevention
WHO	World Health Organization
